# Higher Viral Load of Polyomavirus Type BK but not JC among Renal Transplant Recipients in Comparison to Donors

**DOI:** 10.30699/IJP.2021.535072.2690

**Published:** 2021-12-15

**Authors:** Samaneh Abolbashari, Mohammadtaghi Shakeri, Maryam Hami, Aida Gholoobi, Amin Hooshyar Chechaklou, Mohammasadegh Damavandi, Aref Movaqar, Razieh Yousefi, Zahra Meshkat, Saeedeh Hajebi-Khaniki

**Affiliations:** 1 Student Research Committee, Mashhad University of Medical Sciences, Mashhad, Iran; 2 Department of Medical Genetics and Molecular Medicine, School of Medicine, Mashhad University of Medical Sciences, Mashhad, Iran; 3 Social Determinants of Health Research Center, Mashhad University of Medical Sciences, Mashhad, Iran; 4 Department of Biostatistics, School of Health, Mashhad University of Medical Sciences, Mashhad, Iran; 5 Kidney Transplantation Complications Research Center, Mashhad University of Medical Sciences, Mashhad, Iran; 6 Medical Genetics Research Center, Mashhad University of Medical Sciences, Mashhad, Iran; 7 Antimicrobial Resistance Research Center, Mashhad University of Medical Sciences, Mashhad, Iran; 8 Department of Microbiology, School of Medicine, Isfahan University of Medical Sciences, Isfahan, Iran

## Abstract

**Background & Objective:**

Polyomaviruses types BK and JC and Cytomegalovirus (CMV) have been shown to be related to kidney transplantation complications. This study aimed to assess the prevalence of these viruses in patients receiving kidney transplantation.

**Methods:**

This cross-sectional study was performed on 40 kidney transplant recipients and 44 donors. Urine samples were used for the extraction of viral DNA. The prevalence of JC and BK viruses and their viral loads were determined by real-time polymerase chain reaction.

**Results:**

JC and BK viruses were identified in 31% and 92.3% of all subjects, respectively. The frequency of JC and BK cases was not statistically different between the recipient and donor groups (*P*>0.05). All patients in the donor group and 96.8% of the recipients were positive for CMV IgG antibody. The mean viral load of BK in donors and recipients was 4.5×10^10^ and 3.3×10^11^ copies, respectively. The mean viral load of JC was 8.6×10^7^ copies in donors and 2.9×10^8^ copies in recipients. The distribution of BKV was significantly higher in recipients than donors (*P*=0.001), while no difference was observed between the two studied groups for JCV.

**Conclusion:**

This study showed a relatively high prevalence of BK and JC viruria in both renal transplant donors and recipients. The viral load for BKV, but not JCV, was higher in recipients than in donors.

## Introduction

Among the several types of human polyomavirus identified so far, types BK and JC (initial letters of the names of patients in whom they were first detected) are the major pathogenic viruses (1, 2). The BK virus (BKV) was first discovered in the urine of a renal transplant patient with postoperative ureteral stenosis (3). How-ever, the JC virus (JCV) was initially isolated from a patient diagnosed with Hodgkin’s lymphoma and progressive multifocal leukoencephalopathy (4). Both BKV and JCV can be transmitted through the respiratory tract, fecal-oral route, blood transfusion, and organ transplantation (5-7). It is estimated that 70%-90% of adults have antibodies against both BKV and JCV as most individuals become seropositive in their childhood (2). Primary infection is usually asymptomatic or associated with mild upper respiratory symptoms (1, 8).

After a primary infection, BKV remains latent mainly in the reno-urinary tract. It continues to exist in the urinary tract of the individual and is shed in the urine asymptomatically (9, 10). The strong immunesupp-ression brought about after renal transplantation causes the latent polyomaviruses to reactivate in the kidney and destruct the renal tubular epithelial cells which are associated with the functional impairment of the kidneys (2). While the etiology of Polyomavirus-associated nephropathy (PVAN) is BKV in most cases, JCV has also been associated with nephropathy in a minor group of kidney transplant recipients (11, 12). 

In renal transplant recipients, the prevalence of BKV nephropathy (BKVN) is estimated to be 1%-10% which can result in the rejection of renal transplantation in 40%-80% of cases. The presence of the JC virus in the urine of healthy subjects, in renal transplant recipients, and in liver transplant recipients was 34%, 22.3%, and 22.7%, respectively (13). On the other hand, human Cytomegalovirus (CMV) infection is a frequent disease among renal transplant recipients and can cause adverse consequences (14). 

The relatively high prevalence of this infection has been reported in early childhood, with a proven role in post-transplant complications. Therefore, it is important to determine the prevalence of BK, JC, and CMV infections in renal transplant recipients in order to provide appropriate treatments and prevent adverse effects. Due to the limited studies reported on the prevalence of these viral infections in donors and recipients in Iran, and considering the importance of investigating their role in nephropathy, we measured their prevalence in renal transplant donors and recipients in Khorasan province Iran.

## Material and Methods

This cross-sectional study was implemented on 40 kidney transplant recipients and 44 donors in Montaseriyeh Organ Transplantation Hospital in Mashhad, Iran during 2018-2019. Informed consent was obtained from all subjects. Paired urine and blood samples from patients and donors were collected. First-morning urine samples (counts of epithelial cells were significantly higher in the first voided samples) from patients were taken in the morning. Immediately or within a few hours of keeping the samples in a refrigerator, 15 mL was taken from each urine sample, and after centrifugation for 7 min at 3500 rpm, 300-400 μL of the sediments were deposited into 1.5 mL microtubes. Afterwards, they were transferred to the Microbiological Resistance Research Center of Mashhad University of Medical Sciences for storage at -70°C until real-time polymerase chain reaction (PCR) was carried out. 

DNA extraction from urine was carried out using the Exgene cell SV Kit (GeneAll, Germany) according to the manufacturer’s instructions. Detection and quantification of BK and JC viruses were performed utilizing the BK RQ and JC RQ kits (Novin Gene, Iran), respectively. Virus copies of more than 100 copies/mL were considered positive. PCR ampli-fication was conducted on a Rotor-gene Thermocycler as follows: 95ºC for 10 min, 45 cycles of 95ºC for 15 sec, and 60ºC for 1 min.

The seroprevalence of CMV was determined using an enzyme-linked immunosorbent assay (ELISA) Kit (Pishtazteb, Iran) on serum samples. Moreover, serum specimens were used to assess hematologic indices by a Sysmex autoanalyzer system (KX-21 N). All study participants were also tested for hepatitis C virus (HCV), human immunodeficiency virus (HIV), human T-lymphotropic virus (HTLV-1), HTLV-2, and Epstein-Barr virus (EBV) infections using ELISA commercial kits according to the manufacturer's recommendations. 

**Statistical Analysis**

Descriptive statistics, including frequency (n) and percentage (%) for qualitative variables as well as mean, standard deviation, median, and range for quantitative variables, were calculated separately for the two study groups. For continuous variables, the Shapiro-Wilk test was first applied to determine the normal distribution of data and independent t**-**test or Mann-Whitney test were then used to compare the mean or distribution of the variables between groups. A significance level of 0.05 (α=5%) was adopted and lower P-values were considered significant. All statistical analyses were performed using the SPSS software for Windows version 20.0 (SPSS Inc., Chicago, Ill., USA)**.**

## Results

**Demographic and Clinical Variables**

In the present study, a total of 44 donors and 40 recipients were included. The male:female ratio was 29:15 and 24:16 in donors and those who underwent kidney transplantation, respectively. The difference in the latter ratio was not statistically significant (*P*=0.575). Moreover, there was no statistically significant difference between the two groups in terms of age (*P*=0.219). Regarding the hematologic indices between the two groups, the levels of white blood cells and platelets were significantly higher in donors and recipients, respectively (*P*<0.01). Furthermore, comparing the serum creatinine and urea levels of donors and recipients revealed that the mean serum urea levels were significantly higher (*P*<0.001) in the recipient group, compared to the donors, as were the mean creatinine levels (*P*<0.001). Anti-HCV, anti-HIV, anti-HTLV-1, and anti-HTLV-2 antibodies, as well as the anti-CMV IgM, were negative in all cases of both groups. One of the individuals in the recipient group was positive for EBV IgM antibody. 

**Prevalence of Polyomaviruses and CMV**

 The JC virus was identified in the urine samples of 26 (31%) subjects in both groups, out of which nine belonged to the recipients' group and 17 to the donors' group. BK viruria was identified in 72 (92.3%) cases, 33 of which were in the recipient group and 39 in the donor group (Table 1). The frequency of JCV and BKV positivity was not statistically different between the case and control groups (*P*>0.05). In addition, all people in the donor group and 96.8% of the individuals in the recipient group were positive for CMV IgG antibody, while all subjects in both groups were CMV IgM negative.

**Table 1:** Frequency of BK, JC and CMV viruses in renal transplant recipients and donors

Variable		Donor	Recipient	Total	P-value
BK virus	Negative	2(4.9%)	4(10.8%)	6(7.7%)	**0.415** ^§^
	Positive	39(95.1%)	33(89.2%)	72(92.3%)	
JC virus	Negative	27(61.4%)	31(77.5%)	58(69.0%)	0.110*
	Positive	17(38.6%)	9(22.5%)	26(31.0%)	
CMV virus	IgG negative	0(0%)	1(3.2%)	1(1.4%)	**0.443** ^§^
	IgG positive	39(100%)	30(96.8%)	69(98.6%)	

^§^ Fisher’s exact test.

* Chi-square test.

**Fig. 1:**
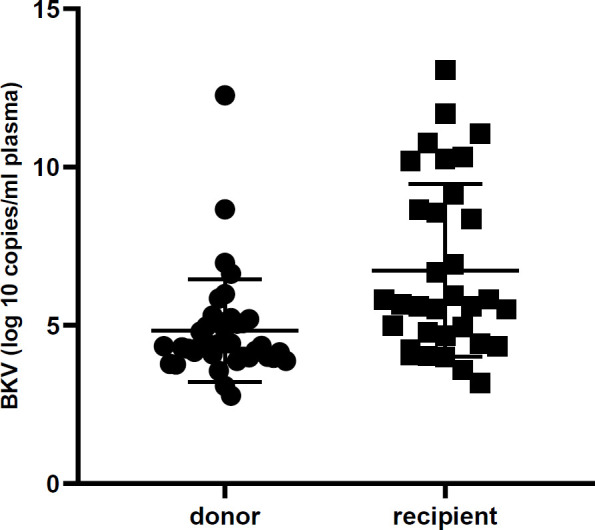
Urine BK virus DNA level in renal transplant recipients and donors. The viral load is significantly higher in the recipient group. (*P*< 0.001)

**Fig. 2:**
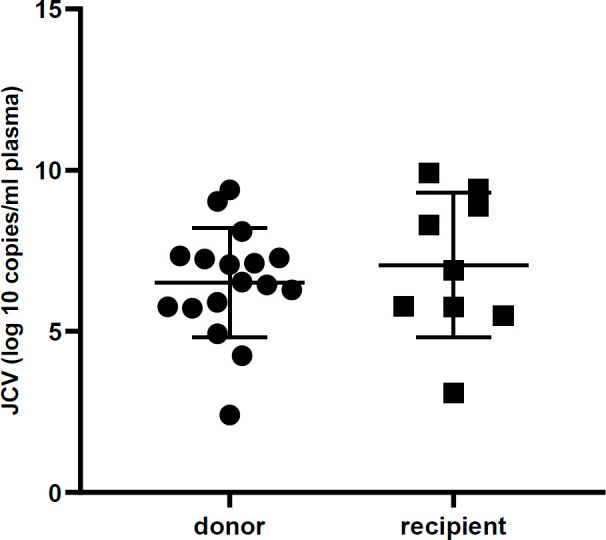
Urine JC virus DNA level in renal transplant recipients and donors. The viral load is not significantly different in the two groups (*P*>0.05)

All urine samples were evaluated by real-time PCR to detect the BKV and JCV DNA and to determine their viral load. The mean viral load of BKV in donors and recipients was 4.5×10^10^ and 3.3×10^11^ copies, respectively. Likewise, the mean viral load of JCV in donors and recipients was 8.6×10^7^ and 2.9×10^8^ copies, respectively. The viral log load of BKV and JCV were compared between donors and recipients, as shown in Table 2. Furthermore, Figures 1 and 2 illustrate the comparison between the viral log load of the two groups. The BKV viral log load was significantly higher in the recipient group than the donor group (*P*< 0.001), while JCV logs viral load did not have any significant difference between donors and recipients (*P*>0.05). As shown in Table 3, the distribution of BKV viral load was significantly different between recipients and donors (*P*=0.001). However, no statistical difference was found between the two studied groups regarding JCV viral load distribution (*P*>0.05). 

**Table 2:** Frequency of BK and JC viruses in renal transplant recipients and donors

Variable		Donor	Recipient	P-value*
BKV (log10 copies/ml plasma)	Mean ± SD	4.8±1.6	6.7±2.7	**0.001**
	Median	4.3	5.6	
JCV (log10 copies/ml plasma)	Mean ± SD	6.5±1.7	7.1±2.2	**0.634**
	Median	6.5	6.9	

* Mann-Whitney test.

**Table 3:** Distribution of the viral load of BK and JC viruses in renal transplant recipients and donors

Variable		Donor	Recipient	Total	P-value*
BK virus viral load	<10^2^	0	0	0	**0.023**
	10^2^-10^3^	1(2.6%)	0	1(1.4%)	
	10^3^-10^4^	8(20.5%)	3(9.1%)	11(15.3%)	
	10^4^-10^5^	18(46.2%)	9(27.3%)	27(37.5%)	
	10^5^-10^6^	8(20.5%)	8(24.2%)	16(22.2%)	
	>10^6^	4(10.3%)	13(39.4%)	17(23.6%)	
	Total	39(100.0%)	33(100.0%)	72(100.0%)	
JC virus viral load	<10^2^	0	0	0	**0.495**
	10^2^-10^3^	1(5.9%)	0	1(3.8%)	
	10^3^-10^4^	0	1(11.1%)	1(3.8%)	
	10^4^-10^5^	2(11.8%)	0	2(7.7%)	
	10^5^-10^6^	3(17.6%)	3(33.3%)	6(23.1%)	
	>10^6^	11(64.7%)	5(55.6%)	16(61.5%)	
	Total	17(100.0%)	9(100.0%)	26(100.0%)	

**Relationship of Polyomaviruses Prevalence with Demographic and Clinical Variables**

According to Table 4, there was no statistically significant relationship between positivity for BK and JC viruses and age or hematology indices, such as WBC, RBC, HB, HCT, MCV, MCH, and MCHC in neither donors nor recipients of renal transplantation (*P*>0.05). There was only a significant difference in the number of platelets, which was lower in recipients positive for the BK virus (*P*=0.02). This difference was not observed in the donor group.

**Table 4:** Relationship between presence of BK and JC viruses and hematologic indices in renal transplant recipients and donors

Variable		Donor							Recipient				
		BK virus			JC virus			BK virus			jc virus		
		Negative	Positive	*P*	Negative	Positive	*P*	Negative	Positive	*P*	Negative	Positive	*P*
Sex	Male	1(50.0%)	27(69.2%)	0.54	15(55.6%)	14(82.4%)	0.10	4(100.0%)	18(54.5%)	0.13	20(64.5%)	4(44.4%)	**0.44**
	Female	1(50.0%)	12(30.8%)		12(44.4%)	3(17.6%)		0	15(45.5%)		11(35.5%)	5(55.6%)	
Age(years)		26±26.8	36.9±19.6	0.41	34.9±21.1	40.3±15.9	0.46	40.5±13.9	32.4±12.8	0.46	30.9±14.5	39.3±9.2	**0.19**
WBC		10.7±4.1	12.7±5.5	0.64	13.1±5.5	12.6±5.0	0.75	11.5±2.9	7.5±3.1	0.08	7.5±2.7	9.1±5.6	**0.85**
RBC		3.7±0.7	4.3±0.8	0.34	4.2±0.9	4.4±0.7	0.45	4.4±0.9	4.2±0.9	0.71	4.2±0.9	3.9±0.4	**0.91**
HB		10.8±2.9	12.7±2.5	0.41	12.1±2.2	13.5±2.7	0.08	12.4±1.1	12.4±2.4	0.80	12.6±2.5	11.6±0.8	**0.63**
HCT		32.7±7.7	38.3±7.0	0.31	35.2±9.0	40.1±6.7	0.09	40.2±4.9	37.5±7.0	0.46	38.7±7.3	34.2±2.4	**0.18**
MCV		87.2±4.1	86.8±6.0	0.85	86.1±6.1	88.9±4.6	0.14	91.5±7.1	89.5±5.5	0.87	90.3±5.6	86.9±3.9	**0.25**
MCH		28.8±2.3	28.4±2.1	0.97	28.2±2.2	29.2±1.6	0.18	28.2±3.0	29.5±2.4	0.64	29.4±2.6	29.1±1.3	**0.50**
MCHC		33.0±1.1	32.8±1.8	0.97	32.9±1.9	32.9±1.3	0.99	30.8±0.9	33±1.6	0.07	32.6±1.7	34.0±1.1	**0.08**
PLT		67.5±13.4	173.6±107	0.10	150.0±85	192.5±127	0.30	265±14.1	181.9±37	0.02	187.4±44.3	196.2±38	**0.57**

*Fisher’s exact test for categorical and Mann-Whitney for quantitative variable.

*P*= P-value

## Discussion

In the current study, the urine samples 31% of the participants were positive for JC virus. We also observed a 92.3% positivity for the BK virus. Although the number of positive cases was higher in the donor group for both viruses, no significant difference was found in the frequency of the two viruses between the recipients and donors. The prevalence we reported is close to that of some western countries but not consistent with what has been reported in other studies conducted in Iran. 

In some European countries, approximately 80% of renal transplant recipients have been reported to have BK viruria (15, 16), which is close to the percentage of BK viruria in our study. However, in a study that assessed the presence of BK virus in 122 renal transplant recipients from southwestern Iran, 41.8% of the urine samples were positive for BKV DNA (17). Another study in Isfahan showed that in renal transplant recipients with kidney dysfunction, 23.3% and 46.7% were positive for JCV and BKV, respectively (18). The BKV is classified into four subtypes and the distribution of different subtypes varies among regions (19). Therefore, the higher incidence found in our study might be related to geographical characteristics and/or the lifestyle of participants and needs for evaluation in larger studies that include numerous factors. 

Unlike our study, which evaluated viral infection using urine samples, some studies have assessed BKV and JCV in serum specimens. A recent study in Shiraz showed that 26.4% of healthy blood donors were positive for BKV DNA in their peripheral blood cells (20). The same investigation reported JC DNA in 18% of blood samples. The prevalence of symptomatic BK infection among transplant recipients of the same hospital was 0.8% during the first year post-transplant (21). An earlier study conducted in Tehran reported BK viremia to be 25% in kidney transplant recipients (22). 

In another study performed on 192 kidney reci-pients, pre-transplantation BKV was positive in the sera of 89 cases (46.35%) (23). In a study conducted in Kashan, Iran, 3.03% of the 33 peritoneal dialysis patients had BK viremia, while none of the 63 hemodialysis patients were positive for BK virus in plasma based on qPCR (24). On the other hand, Jozpanahi *et al.* reported that none of the 50 renal transplant candidates in their study were positive for BK viremia (25). In the assessment of BKV viral load using real-time PCR, we found a higher viral load in recipients than donors. 

Pollara *et al.* detected BKV DNA in the serum samples of 26 out of 75 renal transplant patients by qPCR. The median viral load was 4.1 logs 10 copies/mL in the latter study (26). It has been shown that donor BKV replication is related to post-transplant BKV viremia in the recipient, but it is not associated with urinary BKV replication (27). The risk of evolving a clinically substantial BKV infection is high once a transplant is performed between a positive BKV donor and a negative recipient. As a result, pre- and post-transplantation BKV serology examinations should be taken into account in such cases. However, the usefulness of the mentioned approach is controversial (28). 

We assessed CMV infection in our patients all of whom were positive. This prevalence is higher than what has been reported in another study in Iran. Nasiri *et al.* reported the lack of CMV and BKV coexistence in renal transplant recipients after a month of transplantation. They remark that BKV in neither serum nor urine is associated with CMV in the serum of their patients (29). In a study in Egypt, the seroprevalence of CMV/IgG antibodies in 100 patients undergoing hemodialysis was 98% (30). In addition, Wu *et al.* reported 130 out of 480 samples (27.08%) from renal transplant recipients were positive for CMV DNA (31). Our results are not comparable with the results of the latter study since we have not assessed CMV DNA. In terms of hematologic indices, we found that renal transplant recipients who were positive for BK virus had a lower platelet count in peripheral blood, compared to donors. The reason for this finding needs to be discovered in other larger studies. 

## Conclusion

The present study showed a relatively high shedding of BK and JC viruses in the urine of both renal transplant donors and recipients. The viral load for BKV, but not JCV, was higher in recipients than in donors. Routine evaluation of BKV and JCV is recommended for both transplant recipients and donors. Decreasing the dose of immunosuppressive medications and conducting a longer follow-up in donors are potential suggestions for reducing the adverse effects of viruria in positive cases. This could lead to better transplantation outcomes and may prevent graft rejection.

## Conflict of Interest

The authors declare no conflict of interest.

## Funding

This study was supported by the National Institute for Medical Research Development (NIMAD), Islamic Republic of Iran.
